# Sorbate metal complexes as newer antibacterial, antibiofilm, and anticancer compounds

**DOI:** 10.1186/s12866-024-03370-w

**Published:** 2024-07-18

**Authors:** Amira I. Abousaty, Fifi M. Reda, Wessam A. Hassanin, Walaa M. Felifel, Walaa H. El-Shwiniy, Heba M. R. M. Selim, Mahmoud M. Bendary

**Affiliations:** 1 Botany and Microbiology Department, Faculty of Science, Zagazig University, Zagazig, 44519 Egypt; 2 Department of Chemistry, College of Science, University of Bisha, 61922 Bisha, Saudi Arabia; 3 Department of Chemistry, Faculty of Science, Zagazig University, Zagazig, 44519 Egypt; 4 Department of Pharmaceutical Sciences, College of Pharmacy, AlMaarefa University, P.O. Box 71666, Riyadh, 11597 Saudi Arabia; 5 Microbiology and Immunology Department, Faculty of Pharmacy (Girls), Al-Azhar University, Cairo, 35527 Egypt; 6 Department of Microbiology and Immunology, Faculty of Pharmacy, Port Said University, Port Said, 42526 Egypt

**Keywords:** Sorbic acid, Metal complexes, Biofilm, Anticancer, MTT

## Abstract

**Background:**

The ineffectiveness of treatments for infections caused by biofilm-producing pathogens and human carcinoma presents considerable challenges for global public health organizations. To tackle this issue, our study focused on exploring the potential of synthesizing new complexes of Co(II), Cu(II), Ni(II), and Zn(II) with sorbic acid to enhance its antibacterial, antibiofilm, and anticancer properties.

**Methods:**

Four novel complexes were synthesized as solid phases by reacting sorbic acid with Co(II), Cu(II), Ni(II), and Zn(II). These complexes were characterized by various technique, including infrared spectra, UV–Visible spectroscopy, proton nuclear magnetic resonance (1H NMR), and thermal analysis techniques, including thermogravimetry (TG).

**Results:**

The data acquired from all investigated chemical characterization methods confirmed the chemical structure of the sorbate metal complexes. These complexes exhibited antibacterial and antibiofilm properties against both Gram-positive and Gram-negative bacteria. Furthermore, these complexes enhanced the antibacterial effects of commonly used antibiotics, such as gentamicin and imipenem, with fractional inhibitory concentration (FIC) indices ≤ 0.5. Notably, the Cu(II) complex displayed the most potent antibacterial and antibiofilm activities, with minimum inhibitory concentration (MIC) values of 312.5 µg/mL and 625.0 µg/mL for *Bacillus cereus* and *Escherichia coli*, respectively. Additionally, in vitro assays using the methyl thiazolyl tetrazolium (MTT) method showed inhibitory effects on the growth of the human colon carcinoma cell line (HCT-116 cells) following treatment with the investigated metal complexes. The IC50 values for Co(II), Cu(II), Zn(II), and Ni(II) were 3230 µg/mL, 2110 µg/mL, 3730 µg/mL, and 2240 µg/mL, respectively.

**Conclusion:**

Our findings offer potential for pharmaceutical companies to explore the development of novel combinations involving traditional antibiotics or anticancer drugs with sorbate copper complex.

**Supplementary Information:**

The online version contains supplementary material available at 10.1186/s12866-024-03370-w.

## Background

The efficacy of traditionally used antibacterial drugs has progressively declined owing to the evolution of antibacterial resistance phenomena [1]. The 2022 Global Antimicrobial Resistance and Use Surveillance System (GLASS) report underscored various warning signs concerning the wide-spreading of multi-drug resistant (MDR) pathogens. This crisis significantly impacts national economies and healthcare systems worldwide. Notably, human activities such as the overuse and misuse of antimicrobial drugs have been linked to the emergence and dissemination of resistant pathogens [1]. Furthermore, multiple sectors, including food production, human health, animal husbandry, and the environment, contribute to this issue. It is noteworthy that antimicrobial drug resistance is a natural process driven by microbial genetic changes. These genetic alterations can affect target sites, efflux pumps, or modify the chemical structure of antimicrobial drugs through the production of inactivating enzymes. As newer antibacterial agents are discovered, resistance mechanisms have evolved inconsistently. Traditional antibacterial drugs, which are often targeted by antibiotic-modifying enzymes, can be replaced by newer antibacterial drugs, especially through combination therapies [2]. Even in the presence of these newer therapies, clinicians continue to face significant challenges in treating biofilm-producing pathogens, as biofilm formation remains a persistent problem. Stress conditions, including exposure to physical agents like UV light or changes in hydration or salinity, minimally affect the physicochemical resilience of microorganisms within biofilms [3]. Interestingly, bacterial biofilm is characterized as a multicellular community encased in a polymeric matrix, composed of substances like polysaccharides (alginate), proteins (fibrin), and extracellular genomic DNA, facilitating both bacterial cell clustering and adherence to surfaces. This matrix enables bacteria to enter a dormant state, evade host immune responses, adapt to harsh environmental conditions, and enhance their resistance to antibacterial agents [3]. Similarly, biofilm refers to an organized consortium of microorganisms residing within an extracellular polymer matrix, capable of adhering to both living and non-living surfaces. Once a biofilm forms, bacterial cells exhibit resilience to various bactericidal agents and evade both innate and adaptive immunity [4].

In food production environments, the global burden of foodborne infections is a significant concern, with biofilms capable of forming on virtually all surfaces, leading to food contamination [5]. Consequently, the persistence of biofilms on these surfaces poses a substantial risk to food safety, increasing the likelihood of foodborne illness outbreaks and negatively impacting public health [3]. In the food industry, various environmental factors such as temperature, pH levels of food substrates, and the presence of residual substances significantly influence the formation of biofilms [6]. These factors can enhance the resilience of attached microbial cells to disinfection protocols, rendering conventional cleaning procedures ineffective in eliminating biofilms from food processing environments [6]. Additionally, the ability of pathogenic bacteria such as *Escherichia coli* (*E. coli*) and *Bacillus cereus* (*B. cereus*) to form biofilms has been well-documented, contributing to their pathogenicity and leading to numerous foodborne outbreaks [7]. Quorum sensing, a microbial communication system that regulates bacterial behavior, including the expression of resistance genes, has been implicated in microbial resistance mechanisms. Notably, quorum sensing plays a crucial role in intercellular communication during biofilm formation and facilitates the production of signaling molecules that promote increased cell density within the extracellular polysaccharide matrix [8]. Targeting microbial quorum sensing pathways as well as biofilm production holds promise as a strategy to disrupt microbial resistance mechanisms, especially in food production sectors [9].

On the other hand, cancer is characterized by the uncontrolled proliferation of cells and tissues, resulting in the formation of tumor masses with the potential to metastasize to other regions of the body. As reported by the World Health Organization (WHO), colorectal cancer was the third leading cause of mortality among individuals diagnosed with common cancers, accounting for 1.80 million cases in 2018 [10]. In the United States alone, there were over 100,000 new cancer cases and 50,000 related deaths reported in 2021 [11]. The prevalence of life-threatening conditions such as cancer underscores the urgent need to explore novel sources of potent compounds with anticancer properties. Clinicians face challenges posed by the inherent resistance of biofilm-forming bacteria to conventional antibiotics, as well as the limited responsiveness of cancer cells to chemotherapy. Consequently, treatment failures are common occurrences in managing infections associated with biofilms and human cell carcinoma.

The exploration of novel antimicrobial, antibiofilm, and anticancer compounds has garnered significant attention from our research group. Traditional sources for such compounds have been extensively exploited, leading to a scarcity of new discoveries. Surprisingly, metal complexes formed with natural or synthetic chemical compounds have received relatively little attention [12]. While metal-containing compounds have been utilized in medicinal chemistry since the twentieth century, progress in this area has been gradual. Several bismuth and silver-containing compounds are currently employed as antimicrobial agents. Numerous organic complexes, including organotin, organyltellurium (IV), heterocyclic hydrazine, and thiosemicarbazone ligands, have demonstrated both anticancer and antibacterial properties [13–15]. However, many potentially effective metal-containing compounds remain unexplored. Resistance to metal-containing compounds among biofilm-producing pathogens has been limitedly observed due to their ability to generate reactive oxygen species, which induce damage to various cellular targets such as DNA, RNA, and proteins, alongside their multi-modal mechanisms of action [16].

Despite successful approaches in other areas, the development of organometallic compounds as antibacterial agents has been slow, largely due to limited interest from pharmaceutical companies, despite the diminishing effectiveness of many conventional antimicrobial drugs. Organometallic complexes, such as sorbate metal complexes, have not yet been thoroughly evaluated for their effectiveness against both cancer cells and biofilm-producing pathogens. The choice of sorbic acid (SA) for research into antimicrobial and anticancer activities likely stems from several factors. SA and its derivatives have been previously studied for their potential pharmacological properties due to their structural features and functional groups that could interact with biological systems [17]. Additionally, SA is known for its antimicrobial properties, making it a promising candidate for investigating its potential as an antimicrobial agent. Furthermore, the ability of SA to form metal complexes could enhance its biological activities, making it an intriguing subject for research in both antimicrobial [18] and anticancer applications. The availability and ease of synthesis of SA and its derivatives may have also influenced the author's decision to use it as the focus of their research.

SA, particularly its salt derivative (potassium sorbate), is extensively utilized in food preservation due to its remarkable solubility and stability [17]. Both potassium sorbate and SA find applications in the pharmaceutical industry and food preservation, typically within a concentration range of 0.1–0.2% [19]. Metals like cobalt (Co), copper (Cu), nickel (Ni), and zinc (Zn) can participate in the formation of complexes with SA. Hence, this study aimed to investigate the impact of complexes formed between SA and Co (II), Cu (II), Zn (II), and Ni (II) alone, as well as in combination with commonly used antibacterial drugs, on biofilm-producing foodborne pathogens such as *B. cereus* and *E. coli*. Additionally, the study sought to assess the effects of these complexes on human carcinoma cell lines.

## Methodology

### Materials

The materials used in creating the complexes were all analytical reagents obtained without further purification. Specifically, CoSO_4_.7H_2_O (99.98%, Aldrich Chemical Co.), CuSO_4_.5H_2_O (99.9%, Aldrich Chemical Co.), ZnSO_4_ (99.9%, Fluka Chemical Co.), NiCl_2_.6H_2_O (99.9%, Fluka Chemical Co.), and SA (99.98%, Aldrich Chemical Co.) were used in this study. Additionally, all solvents utilized were sourced from Merck Ltd. and Sigma Aldrich and were stored in tightly sealed containers to prevent environmental evaporation.

### Instruments

The FT-IR 460 PLUS Spectrophotometer was used, with potassium bromide plates, to capture IR spectra in the range of 4000–400 cm-1. Proton nuclear magnetic resonance spectra were acquired using DMSO-d6 as the solvent on a Varian Mercury VX-300 NMR Spectrometer. Electronic spectra were obtained using the Shimadzu UV-3101PC. Absorption spectra were recorded as DMSO-d6 solutions. The Shimadzu TGA-50H was used for implementation, with TG-DTG measurements conducted in an N2 environment at temperatures ranging from 0 °C to 1000 °C, with the sample mass precisely measured in an aluminum crucible. The M percent content was determined using three analytical methods: complexometric titration, thermogravimetry involving the conversion of solid products into metal oxides, and atomic absorption spectroscopy using a PYE-UNICAM SP 1900 spectrometer equipped with the appropriate lamp. Elemental analysis was carried out using a Perkin Elmer 2400 CHN elemental analyzer. The melting points were determined using Buchi equipment and are reported without correction. Magnetic susceptibilities of the powdered materials were measured at room temperature using a Gouy balance, a Sherwood Scientific magnetic scale, with Hg[Co(CSN)4] as the standard. Molar conductance of 1 × 10–3 M solutions of ligands and their complexes in DMF was analyzed using CONSORT K410. All experiments were conducted with freshly prepared solutions, and their progress was monitored using thin-layer chromatography (TLC) at room temperature.

### Synthesis of sorbate metal complexes

The synthesis of the white solid complex [Co(SA)2(H2O)2] was achieved by dissolving 2 mmoL (0.222 g) of SA and 2 mmoL (0.08 g) of sodium hydroxide (NaOH) in 30 mL of ethanol. Subsequently, 0.281 g (1 mmoL) of cobalt sulfate heptahydrate (CoSO4.7H2O) in 10 mL of ethanol was gradually added to the reaction mixture and refluxed for 6 h. After allowing the mixture to evaporate slowly at room temperature for several days, the precipitate was separated and vacuum-dried over anhydrous calcium chloride (CaCl2). Additionally, pale green [Ni(SA)2(H2O)2], blue [Cu(SA)2(H2O)2], and off-white [Zn(SA)2(H2O)2] solid complexes were synthesized using nickel chloride hexahydrate (NiCl2.H2O), copper sulfate pentahydrate (CuSO4.5H2O), and zinc sulfate (ZnSO4), respectively, with a molar ratio of 2:1 (SA:M) in ethyl alcohol [20, 21].

#### Analytical data of the compounds

1. Sorbic acid (SA) ligand
  White; m.p.: 134.53 ℃; M.Wt: 111.13; Elemental analysis for C6H8O2: found, C, 63.50; H, 7.16; Calcd, C 64.27; H, 7.19; Λm = 12.0 S cm2 mol−1; IR (KBr, v, cm−1): 3412 m,br (ν(O–H), COOH), 1615s (ν(C = O); COOH). 1H NMR (DMSO-d6, 300 MHz): δ = 2.05 (s, 3H, CH3), 5.34–7.47 (S, 4H, = CH), 12.05 (s, 1H, -COOH).
2. [Co(SA)2(H2O)2] complex
  White; Yield: 80.03%; m.p.: 183.22 ℃; M.Wt: 377.17; Elemental analysis for CoC12H14O10: found, C, 38.62; H, 3.52; Co, 15.43; Calcd, C 38.21; H, 3.52; Co, 15.63; Λm = 19.0 S cm2 mol−1; IR (KBr, v, cm−1): 3417 m,br (ν(O–H), H2O), 1562 (νas(COO−)), 1423 (νs(COO−)) and 460 (ν(M–O). 1H NMR (DMSO-d6, 300 MHz): δ = 2.49 (s, 3H, CH3), 3.46 (s, 2H, H2O), 5.14–6.97 (s, 4H, = CH).
3. [Ni(SA)2(H2O)2] complex
  Pale green; Yield: 77.23%; m.p.: 288.31 ℃; M.Wt: 376.93; Elemental analysis for NiC12H14O10: found, C, 38.12; H, 3.72; Ni, 15.54; Calcd, C, 38.24; H, 3.74; Ni, 15.57; Λm = 17.0 S cm2 mol−1; IR (KBr, v, cm−1): 3426 m,br (ν(O–H), H2O), 1562 (νas(COO−)), 1423 (νs(COO−)) and 590 (ν(M–O). 1H NMR (DMSO-d6, 300 MHz): δ = 2.39 (s, 3H, CH3), 3.75 (s, 2H, H2O), 5.50–7.77 (s, 4H, = CH).
4. [Cu(SA)2(H2O)2] complex
  Blue; Yield: 82.11%; m.p.: 186.66 ℃; M.Wt: 321.81; Elemental analysis for CuC12H18O6: found, C, 44.19; H, 5.34; Cu, 19.16; Calcd, C 44.79; H, 5.64; Cu, 19.75; Λm = 10.2 S cm2 mol−1; IR (KBr, v, cm−1): 3419 m,br (ν(O–H), H2O), 1558 (νas(COO−)), 1411 (νs(COO−)) and 520 (ν(M–O). 1H NMR (DMSO-d6, 300 MHz): δ = 2.33 (s, 3H, CH3), 3.65 (s, 2H, H2O), 5.44–7.17 (s, 4H, = CH).
5. [Zn(SA)2(H2O)2] complex
  Off-white; Yield: 85.91%; m.p.: 210.61 ℃; M.Wt: 323.68; Elemental analysis for ZnC12H18O6: found, C, 44.23; H, 5.08; Zn, 20.11; Calcd, C 44.53; H, 5.61; Zn, 20.21; Λm = 15.5 S cm2 mol−1; IR (KBr, v, cm−1): 3451 m,br (ν(O–H), H2O), 1558 (νas(COO−)), 1442 (νs(COO−)) and 565 (ν(M–O). 1H NMR (DMSO-d6, 300 MHz): δ = 2.43 (s, 3H, CH3), 3.88 (s, 2H, H2O), 5.15–7.66 (s, 4H, = CH).

## Isolation of *Bacteria* from Food Samples

### Food Samples

Various food products, including chickens, pickles, cheeses, fish, and milk samples, were collected from different markets in Zagazig, Egypt, from March to September 2017. These collected samples were utilized for the isolation of pathogenic bacteria.

### Isolation of Pathogenic *Bacteria*

Ten grams of each sample were mixed with 90 mL of sterile saline solution (NaCl,0.85%) and agitated for 2 min. Decimal dilutions were prepared, and 0.1 mL of each dilution was spread onto the surface of prepared nutrient agar plates. The resulting colonies were purified and preserved on nutrient agar slants (Difco-Bacto, Carolina, USA) at 4°C.

## Estimation of Biofilm production

### Qualitative congo red agar method

The direct detection of biofilm-producing strains was conducted in triplicate using the Congo Red Agar (CRA) method [22]. The bacterial isolates were cultured in Brain Heart Infusion (BHI) broth (DIFCO®) supplemented with 0.08% Congo red and 5% sucrose, followed by incubation at 37°C for 24 h. After incubation, black colonies with a dry crystalline consistency on the red agar indicated biofilm production, resulting from the interaction between surface biofilm proteins and Congo red dye. Conversely, non-biofilm producers appeared as smooth, red colonies [23]. This method exhibited high specificity (no false positive results) and low sensitivity (no false negative results), thus rendering the CRA method reliable for detecting biofilm-producing isolates[24]. Additionally, biofilm and non-biofilm producing Methicillin-resistant *Staphylococcus aureus* (MRSA) strains obtained from the Animal Production Research Institute, Giza, Egypt, were utilized as positive and negative controls, respectively.

### Quantitative microtiter plate test

The microtiter plate (MTP) test was employed to quantitatively assess biofilm production by detecting the optical density of stained isolates using a spectrophotometer. Ninety-six flat-bottom tissue culture plates were used for biofilm formation evaluation [25]. Overnight bacterial suspensions were prepared in BHI broth with 1% glucose, further diluted with fresh medium (1:100), and 200 μL of bacterial suspension was inoculated into each well, incubated at 37 °C for 48 h. After incubation, the plate wells were gently aspirated and washed with 200 µl of 1X phosphate buffer saline (PBS) (pH 7.2). Biofilms adherent to the well walls and bottoms were fixed with 2% sodium acetate and stained with 2% crystal violet at room temperature. Excess stain was removed with deionized water, and plates were properly dried. It is worth mentioning that biofilm and non-biofilm producing MRSA strains were used as positive and negative controls, respectively. The optical densities (OD at 570 nm) of stained adherent biofilms were obtained with a micro-ELISA auto reader (model) located at the Central Laboratory, Faculty of Pharmacy, Port Said University. Biofilm assays for each bacterial isolate were carried out in triplicate, and their mean absorbance values were determined and distinguished as strong (> 0.240) and moderate (0.120 to 0.240). No biofilm formers were detected below 0.05 based on their OD values at 570 nm [26].

### Identification of species employing both phenotypic and genotypic detection techniques

The biofilm-producing bacteria were identified phenotypically based on their colonial morphologies, culture characteristics, cell shapes, and biochemical reactions [27]. Confirmation of identification was achieved through 16S rRNA gene sequencing, as it was previously described [28]. The obtained 16S rRNA sequences were deposited on the NCBI web server for further analysis. Sequence analysis and comparison with published sequences were conducted using the Basic Local Alignment Search Tool (BLAST) program sequence-matching (https: // www. ncbi. nlm. nih.gov/blast).

## Determination of antibacterial activity of sorbate metal complexes

### Agar well diffusion method

The antibacterial activities of the tested complexes against foodborne biofilm-producing bacterial isolates were assessed in triplicate using the agar well diffusion method. Stock solutions of each tested complex were prepared by dissolving 0.1 g of the compound in 10 mL of dimethylformamide (DMF). The selected biofilm-producing strains were inoculated onto agar plates, and wells were created. Subsequently, 100 µL of each complex solution (200 µg/mL) was added to the wells, with positive and negative control wells containing standard antibiotics (gentamicin) and DMF, respectively, also included. After incubation at 37°C for 24 h, the diameter of the growth inhibition zones was measured [29].

### Minimum Inhibitory Concentrations (MICs) and Minimum Bactericidal Concentrations (MBC) by Microdilution Method

The MICs of the tested compounds were determined, in triplicate, using the standard broth microdilution technique. Two-fold serial dilutions of each compound were prepared, ranging from 0 to 5000 µg/mL, in Mueller–Hinton broth. The dilutions were added to 96-well microtiter plates containing the prepared microbial suspensions. Positive and negative control wells were included to validate growth conditions. Following incubation at 37°C for 24 h, the growth of the isolates was assessed by measuring turbidity at an optical density of 620 nm using an ELISA microplate reader [30]. The MIC was defined as the lowest concentration of the tested compound that inhibited visible growth, while the MBC was defined as the lowest concentration that resulted in no visible growth after sub-culturing on fresh medium [31].

### Assessment of interaction between sorbate metal complexes and commonly used antibacterial drugs via checkerboard assay

The interaction between Sorbate metal complexes and commonly used antibacterial drugs (gentamicin and imipenem) was evaluated using a checkerboard assay in triplicate, following a previously published protocol [32]. The fractional inhibitory concentration index (FICI) was calculated to determine the interaction results. The FICI was computed using the equation FICI = FIC(A) + FIC(B), where FIC(A) represents the MIC of Sorbate metal complexes in combination divided by the MIC of Sorbate metal complexes alone, and FIC(B) represents the MIC of the antibacterial drug in combination divided by the MIC of the antibacterial agent alone. Interpretation of the FICI values indicated additivity (0.5 < FICI ≤ 1), antagonism (FICI > 4), synergism (FICI ≤ 0.5), and indifference (1 < FICI ≤ 4).

### Antibiofilm activity of sorbate complexes

The inhibition of biofilm formation by metal complexes against biofilm-producing foodborne isolates was investigated using the crystal violet staining assay in a microtiter plate assay [33]. Sub-MIC concentrations (0.5 MIC) of each sorbate metal complex were used to evaluate their anti-biofilm activities. The test was repeated three times, with both positive and negative controls included in each repetition. The wells of microtiter plates were filled with 1 ml of an overnight culture of each tested strain (106 CFU/mL), followed by the addition of 1 ml of the sub-MIC of tested metal complexes to each well. The plates were then incubated at 37°C for 48 h, and biofilm formation was assessed using an ELISA microplate reader. Two culture wells were tested: one devoid of metal complexes served as the positive control, while the other containing DMF served as the negative control. The percentage of microbial adhesion was calculated in triplicate using the formula: % of Microbial adhesion = (AC/A0) × 100, where AC represents the absorbance of the well with metal complexes, and A0 represents the absorbance of the positive control well [34].

### Cytotoxicity activity of sorbic acid complexes against human carcinoma cell lines

The in vitro cytotoxicity of SA complexes against human carcinoma cell lines was assessed using the methyl thiazolyl tetrazolium (MTT) method as described previously [35]. This colorimetric technique relies on the conversion of a yellow tetrazolium salt, specifically 3-(4,5-dimethylthiazol-2-yl)-2,5-diphenyltetrazolium bromide (MTT), into purple formazan crystals by metabolically active cells. The inhibitory activity of synthesized compounds was compared with a well-known anticancer drug (doxorubicin). The data response curve showed which concentration of the tested compounds is required to kill 50% of the cell population (IC_50_). The used cell lines such as human breast carcinoma cell line (MCF-7 cells), human colon carcinoma cell line (HCT-116 cells), and lung carcinoma cell line (A549 cells) were obtained from the American Type Tissue Culture Unit. The mammalian cells were propagated in Dulbecco’s Modified Eagle’s Medium (DMEM) or RPMI-1640, depending on the type of cell line, supplemented with 10% heat-inactivated fetal bovine serum, 1% L-glutamine, HEPES buffer, and 50 µg/mL gentamycin. All cells were maintained at 37 ºC in a humidified atmosphere with 5% CO_2_ and were sub-cultured two times a week during experimentation [36]. All experiments were conducted in triplicate.

## Results

### Chemistry part

#### Elemental and molar conductance

The results of melting points, molar conductance, m/z values, and elemental analysis for SA and its metal complexes are shown in Table S1. The elemental analytical data matched the estimated values for the molecular formulae used to describe these metals chelates. The complexes are non-hygroscopic and sparingly soluble in water, while they are soluble in DMSO and DMF; they are also stable at room temperature. The molar conductance for complexes was assessed in 1* 10–3 M solutions using DMSO, revealing that all complexes were non-electrolytes. All complexes were formed from the reaction of SA with metal salts in a 2:1 molar ratio.

#### Infrared band assignments

**Fig. 1:**
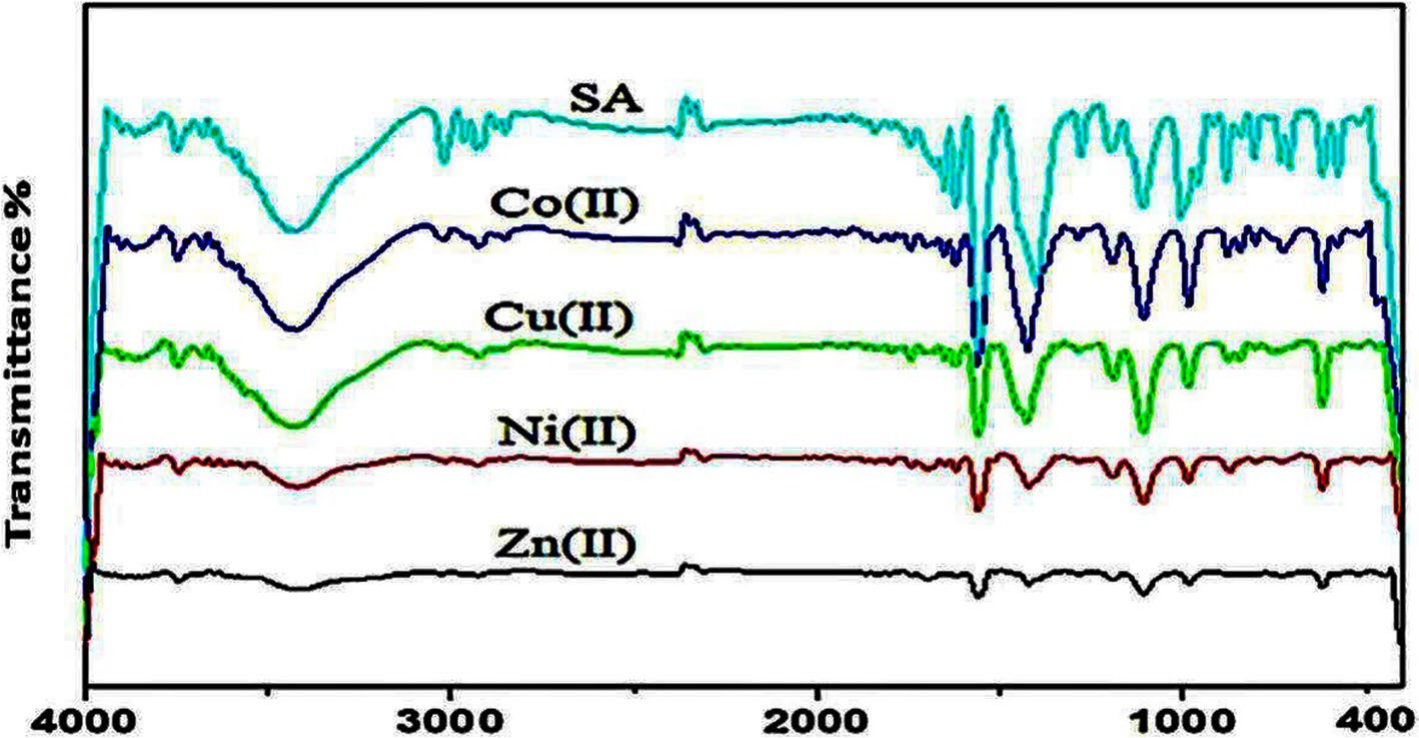
Characterization of sorbate metal complexes using Infrared spectra

**Fig. 2:**
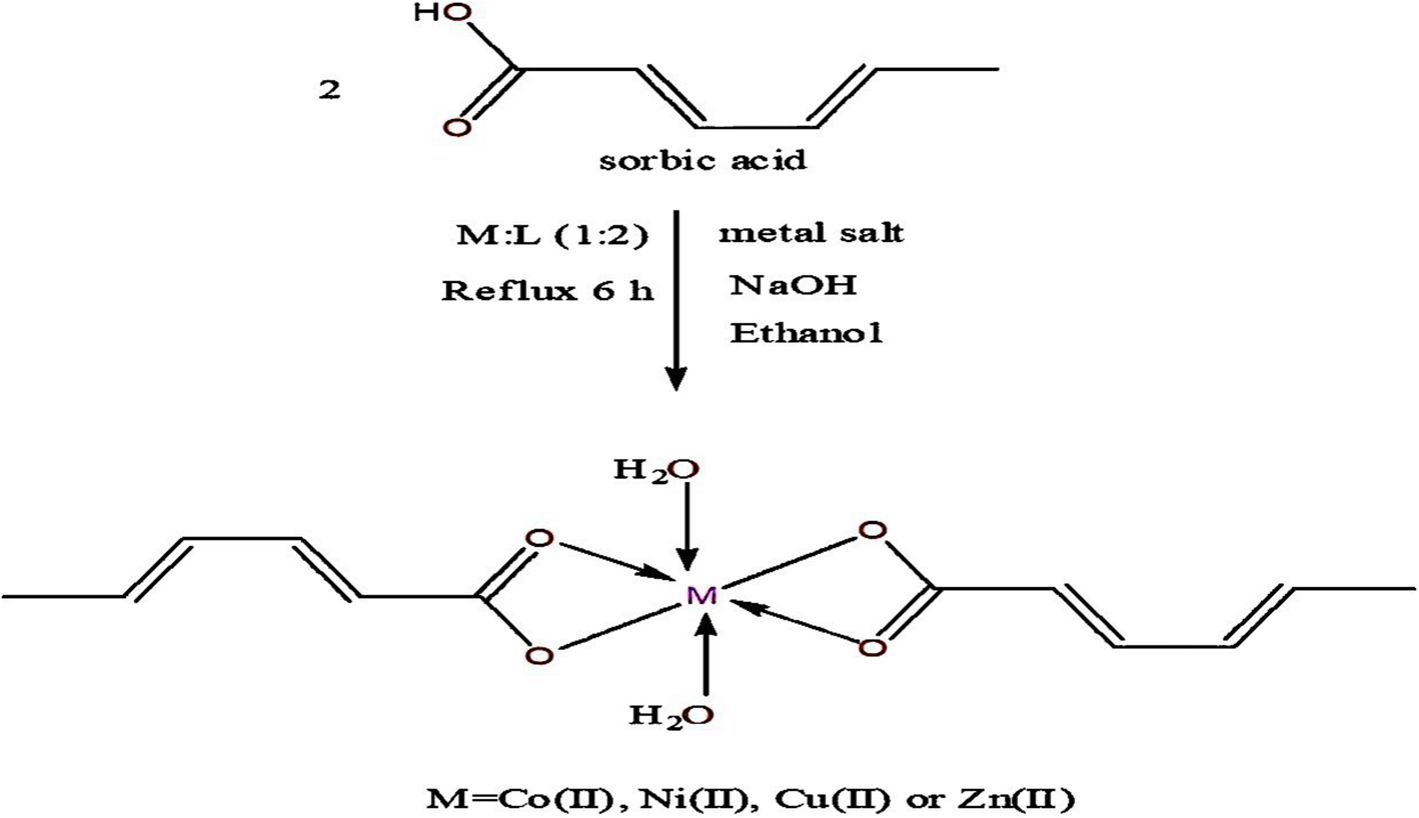
Synthesis of sorbate metal complexes and the suggested complexes structures

IR spectra determined the coordination site, where the anti-symmetric and symmetric COO^−^ stretches were shifted to higher and lower frequencies, respectively, with an average ∆ν of 139 cm^−1^. Most SA complexes had bidentate coordination with an average Δ*ν* of 160 cm^−1^. Coordination via the oxygen of carboxylate was confirmed by *ν*(M–O) bands at 460 cm^−1^ for Co (II), 590 cm^−1^ for Ni (II), 520 cm^−1^ for Cu (II), and 565cm^−1^ for Zn (II), as shown in Fig. 1. As a result, SA was coordinated with metal through the oxygen of carboxylates. The SA complexes demonstrated characteristic IR spectra due to ligand vibration modes. The ν as (COO^−^) and *ν*_s_(COO^−^) for carboxylate depended upon the coordination mode of the carboxylate ligand. Both *ν*(C = O) bonds were equivalent in the free ion, and the anti-symmetric and symmetric COO^−^ stretches appeared at 1651 and 1450 cm^−1^, respectively. The anti-symmetric and symmetric COO^−^ stretches were shifted to higher and lower frequencies with an average ∆*ν* of 139 cm^−1^. If coordination occurred symmetrically, both COO^−^ stretches would be shifted in the same direction since both *ν*(C = O) bonds might be changed by the same amount. Bidentate coordination was found for most SA complexes with an average Δ*ν* value of 160 cm^−1^. Ν (M–O) bands confirmed coordination via oxygen of carboxylate at 460 cm^−1^for Co (II), 590 cm^−1^ for Ni (II), 520 cm^−1^ for Cu (II), and 565 cm^−1^ for Zn (II). Based on IR spectrum, SA was coordinated with metal through the oxygen of carboxylates forming octahedral complexes (Fig. 2).

#### Thermal studies

The thermal stability of water molecules within or outside the coordination sphere was determined by TGA analysis, which examined temperatures ranging from 0 to 800°C. The thermal degradation of the Co(II) complex occurred in three stages (FigureS1 and Table S2). The first stage of decomposition took place at a maximum temperature of 258°C and was accompanied by a weight loss of 9.42%, corresponding to the loss of coordinated water molecules. The second and third decomposition steps occurred at 425°C and 596°C, respectively, with a total weight loss of 70.98%, indicating the decomposition of the organic ligand moiety, leaving CoO as residue (19.60%). Similarly, the Ni(II), Cu(II), and Zn(II) complexes underwent decomposition following the same steps as the Co(II) complex, resulting in the formation of NiO, CuO, and ZnO residues. Therefore, the obtained results demonstrate the stability of the Co(II), Ni(II), Cu(II), and Zn(II) complexes above 258°C, 210°C, 197°C, and 151°C, respectively, confirming the presence of water molecules within the coordination sphere and the non-volatile nature of the complexes. Based on the preceding analysis, the proposed complex structures are illustrated in Fig. 2.

#### Magnetic moment and UV–vis spectra

The UV–vis spectrum of SA was measured between 200 and 800 nm in DMSO (Figure S2 and Table S3). The bands observed at 295 nm, 310 nm, and 385 nm are attributable to π → π* and n → π* transitions. These bands shift to a blue wavelength upon chelation, indicating the involvement of carboxylate oxygen atoms in coordination. All complexes exhibited bands in the range of 423–475 nm, indicative of ligand to metal charge-transfer transitions. In the electronic spectrum of the Co(II) complex, three prominent bands were observed at 19,417 cm^−1^, 16,393 cm^−1^, and 14,814 cm^−1^, corresponding to the ^4^T_1g_(F) → ^4^T_1g_(P), ^4^T_1g_(F) → ^4^A_2g_(F) and ^4^T_1g_(F) → ^4^T_2g_(F) transitions, respectively, with a magnetic moment value of 4.71 B.M. For the Ni(II) complex, the bands were observed at 19,607 cm^−1^, 14,925 cm^−1^, and 14,084 cm^−1^, attributable to the ^3^A_2g_(F) → ^3^T_1g_(P), ^3^A_2g_(F) → ^3^T_1g_(F) and ^3^A_2g_(F) → ^3^T_2g_ transitions, respectively, with a magnetic moment of 3.0 B.M. The Cu(II) complex exhibited a band at 19,607 cm^−1^, corresponding to the ^2^E_g_ → ^2^T_2g_ transition, with a magnetic moment of 1.83 B.M. No d-d transition bands were observed in the diamagnetic Zn(II) complex. The magnetic moments (Table S3) confirmed the octahedral structure of all complexes.

#### ^1^H NMR spectra

^1^H NMR spectra of SA in DMSO-d_6_ show singlets at *δ*: 2.05 ppm corresponding to methyl group; singlets at *δ*: 5.34–7.47 ppm for = CH. The peak at 12.05 ppm can be assigned to carboxylic proton (COOH) (Figure S3). ^1^H NMR spectra of [Co(SA)_2_(H_2_O)_2_], [Ni(SA)_2_(H_2_O)_2_], [Cu(SA)_2_(H_2_O)_2_], [Zn(SA)_2_(H_2_O)_2_] in *DMSO-d*_*6*_ exhibit new resonances at 3.46, 3.75, 3.65 and 3.88 ppm, due to the presence of water in the complexes. The carboxylic proton (COOH) is not detected in spectra of the complexes, suggesting coordination of SA through carboxylate oxygen. On comparing SA with its complexes, all peaks of the free ligand are present in spectra of the complexes with some shifts from binding of the ligand to the metal.

#### Isolation of food samples

Eighty bacterial cultures were isolated from various food product samples, with 34 identified as Gram-positive and 46 as Gram-negative bacterial isolates. The most contaminated food items were meats (25.0%), followed by chickens (18.75%), pickles (6.25%), cheeses (15.0%), fish (13.75%), and milk (11.25%).

#### Identification of biofilm-producing *bacteria*

Congo Red Agar (CRA) plates were utilized to assess the biofilm-forming abilities of the isolated bacteria. Among the 80 bacterial isolates, 71 displayed red smooth colonies, while 9 exhibited dark black colonies with optical densities ranging from 0.411 to 0.385. Thus, only 9 isolates demonstrated the ability to form biofilms. Furthermore, the biofilm activities of these 9 isolates were confirmed using the microtiter plate (MTP) test, with mean absorbance values above 0.240 AU. This confirmed the consistency between the results obtained from CRA and the MTP test.

#### Characterization of selected isolates capable of producing biofilms at a species level

**Fig. 3:**
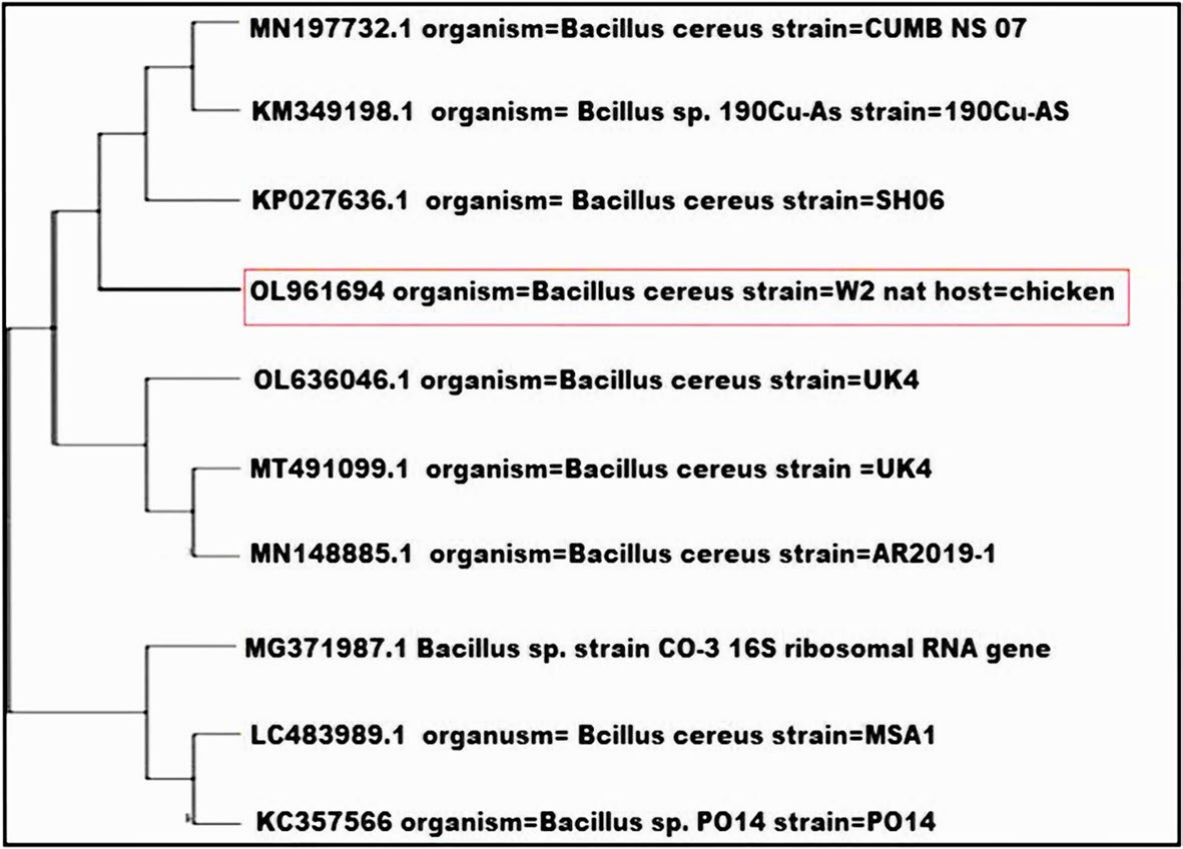
Phylogenetic tree analysis of *B. cereus* strains

**Fig. 4:**
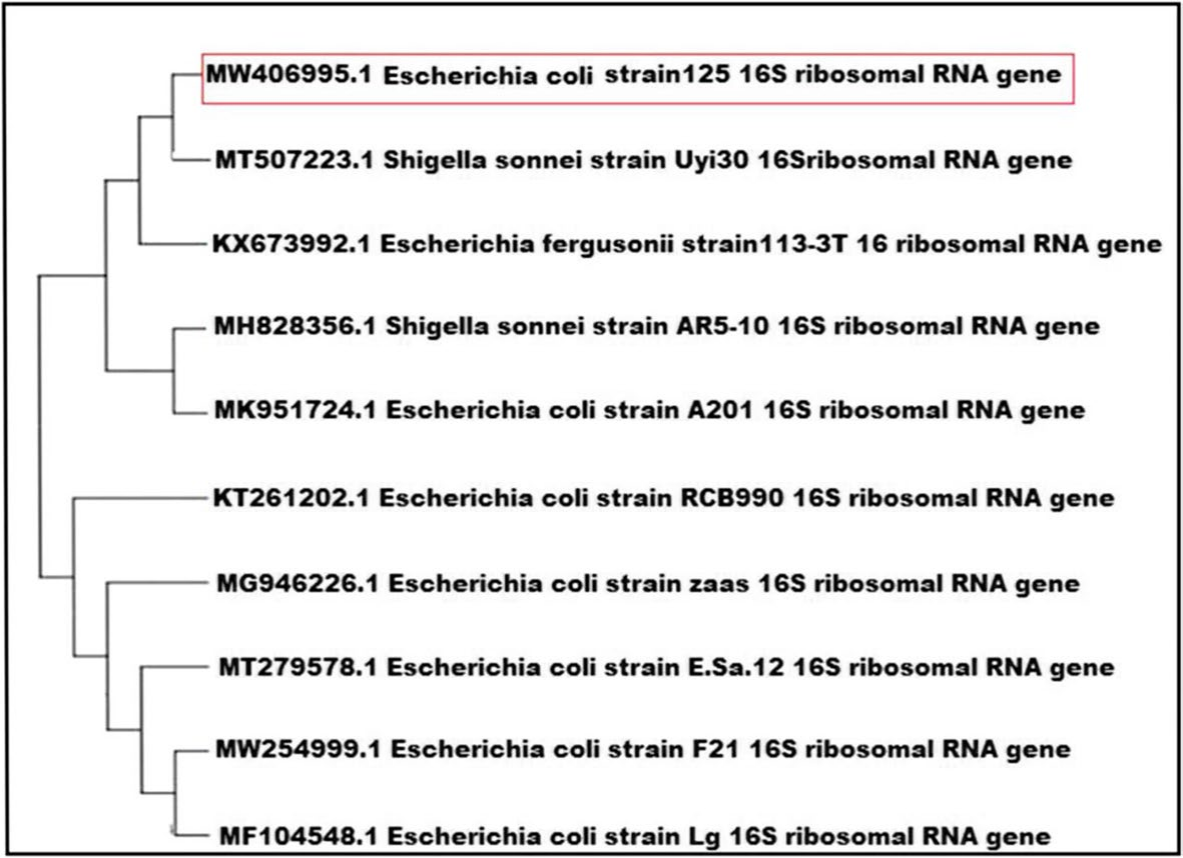
Phylogenetic tree analysis of *E. coli* strains

Based on Bergey's Manual of Determinative Bacteriology and other biochemical tests, the selected biofilm-producing bacterial isolates were identified as 4 strains of *B. cereus* and 5 strains of *E. coli*. The identification of these isolates was further confirmed through PCR amplification and sequencing of the 16S rRNA genes, and the sequences were deposited in the NCBI database server (https://www.ncbi.nlm.nih.gov/genbank) under accession numbers OL961694 and MW406995, respectively (Figs. 3 and 4). The *B. cereus* isolates were found to be distinct from other strains isolated from soil in Eskişehir province, Turkey (KP027636.1), and fish in Egypt (LC483989.1), as well as strains from India (KC357566.1) isolated from *Drosophila ananassae* (Fig. 3). Regarding the *E. coli* isolates, they were closely related to human fecal *E. coli* isolates in Switzerland (KX673992.1) but distantly related to nasal swab *E. coli* isolates in China (MF104548.1) (Fig. 4). This indicates that the food isolates in this study shared significant similarities with isolates from common contamination sources such as soil and sewage, despite geographical differences. However, they exhibited high diversity compared to strains isolated from other sources.

#### Antibacterial activities of sorbate metal complexes on biofilm- producing bacterial isolates

Using the agar well diffusion method, the biofilm-producing bacterial isolates (5 *E. coli* and 4 *B. cereus*) were selected and tested against sorbate metal complexes. Notably, no antibacterial activity was observed for the solvent in the negative control wells; however, higher antibacterial activities were seen in the positive control wells (gentamicin). The *B. cereus* isolates exhibited sensitivity to three complexes, with mean inhibition zone diameters of 27.0 ± 0.33 mm, 29.3 ± 0.82 mm, and 22.0 ± 0.41 mm for Co(II), Cu(II), and Zn(II) complexes, respectively. However, the Ni complex did not show any response. Meanwhile, all tested complexes (Co(II), Cu(II), Zn(II), and Ni(II)) exhibited significant effects on all *E. coli* isolates, with mean inhibition zone diameters of 25.66 ± 0.50 mm, 27.63 ± 0.66 mm, 25.34 ± 0.62 mm, and 24.36 ± 0.83 mm, respectively. Among these complexes, the Cu(II) complex showed the highest efficacy against most bacterial isolates, followed by Co(II), Zn(II), and Ni(II) complexes.

The MIC values of the sorbate metal complexes were determined. For *B. cereus* isolates, the MIC values for Co(II), Cu(II), and Zn(II) complexes were 625.0, 312.5, and 1250.0 µg/mL, respectively, while the MBC values were 625.0, 625.0, and 1250.0 µg/mL, respectively. For *E. coli* isolates, the MIC values were 625 µg/mL for Co(II), 625 µg/mL for Cu(II), 1250 µg/mL for Zn(II), and 1250 µg/mL for Ni(II) complexes. The MBC values for these complexes were 1250, 625, 1250, and 2500 µg/mL, respectively. Generally, Gram-positive bacteria such as *B. cereus* were more sensitive to sorbate metal complexes than Gram-negative pathogens such as *E. coli.*

**Fig. 5:**
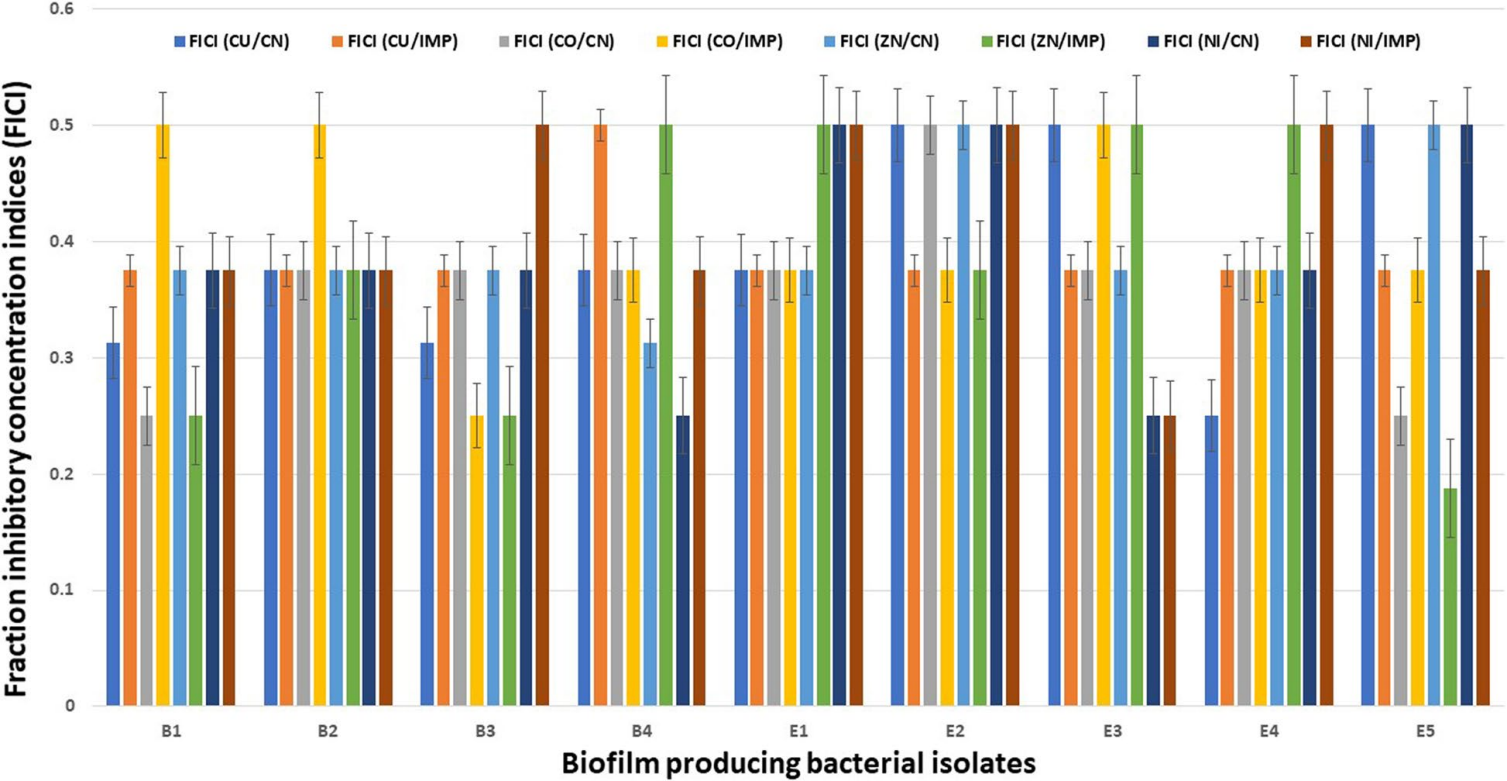
Fractional inhibitory concentration indices (FICI) for the combination of Sorbate metal complexes (CU,CO,ZN,NI) with antibiotics such as gentamicin (CN) and imipenem (IMP)

The in vitro interaction of sorbate metal complexes with commonly used antibacterial drugs such as gentamicin and imipenem was evaluated using a checkerboard technique. The FICI values for both sorbate metal complexes with gentamicin and with imipenem were < 0.5 (Fig. 5 and Tables S4, S5, S6, and S7), indicating synergistic action between these complexes and the commonly used antibacterial drugs. Therefore, sorbate metal complexes can potentiate the antibacterial activities of gentamicin and imipenem against biofilm-producing pathogens.

#### Structure activity relation ship regarding to antimicrobial activities

By comparing the chemical structures of the sorbate complexes of zinc, copper, cobalt, and nickel with their associated biological activities. The structure–activity relationship (SAR) analysis reveals that the copper complex exhibits the highest antimicrobial activity against both Gram-positive and Gram-negative bacteria, as evidenced by the lowest minimum inhibitory concentration (MIC) values. This superior antimicrobial efficacy is attributed to the unique electronic configuration and redox properties of copper, which facilitate stronger interactions with microbial cell proteins, leading to enhanced disruption of essential biological processes. Additionally, higher ability of copper to participate in Fenton-like reactions generates reactive oxygen species (ROS) that can damage cellular membranes, proteins, and DNA of the bacteria. The relatively lower activity observed in zinc, cobalt, and nickel complexes suggests that these metals either interact less effectively with microbial target proteins or generate fewer reactive oxygen species (ROS), highlighting the pivotal role of metal ion properties in determining the antimicrobial effectiveness within this complex series. Hence, the electron configurations of these metals determine their chemical reactivity, coordination preferences, and capability to engage in redox reactions, all of which can impact the biological activities demonstrated by their sorbate complexes.

#### Antibiofilm activity of sorbate metal complexes

The influence of sorbate metal complexes on biofilm production was evaluated across various strains, encompassing both Gram-positive and Gram-negative bacteria. The findings revealed a consistent trend: all sorbate metal complexes correlated with reduced biofilm formation across all tested strains. Notably, the copper complex exhibited exceptional efficacy, resulting in only 8.7% biofilm adhesion for *B. cereus* isolates and 17.3% for *E. coli* isolates (Table 1). Overall, the results underscore the considerable potential of sorbate metal complexes in impeding biofilm formation, with varying effectiveness depending on the specific metal complex and bacterial strain.

**Table 1:** The percentage of microbial adhesion of the treated isolates with sub-MIC concentration of each metal complex

Treated isolates	Co (II)		Cu (II)		Zn (II)		Ni (II)	
	**OD**	**%**	**OD**	**%**	**OD**	**%**	**OD**	**%**
*B. cereus*	0.059 ± 0.001	13.4	0.038 ± 0.002	8.7	0.109 ± 0.5	24.8	0.219 ± 0.5	49.9
*E. coli*	0.107 ± 0.01	24.4	0.076 ± 0.002	17.3	0.134 ± 0.001	30.5	0.147 ± 0.002	33.5

*OD* Optical density, Control OD = 0.439

The observed relationship between structure and activity emphasizes the outstanding antibiofilm capabilities of the copper sorbate complex compared to its cobalt, nickel, and zinc counterparts across both Gram-positive and Gram-negative bacterial strains. This superiority suggests that specific structural characteristics or chemical properties inherent to the copper complex are crucial in hindering biofilm formation. The unique coordination environment or electronic configuration of copper ions within the sorbate complex may facilitate enhanced interactions with bacterial cells or components of the biofilm matrix, thereby more effectively impeding biofilm formation compared to other metal complexes. These findings underscore the significance of considering the structural attributes of metal complexes concerning their biological functions, offering valuable insights for the design and enhancement of antibiofilm agents.

#### Cytotoxic activity of sorbate metal complexes

The cytotoxic activity of sorbate metal complexes was evaluated using the MTT colorimetric assay on three human cell lines: human breast carcinoma cell line (MCF-7 cells), human colon carcinoma cell line (HCT-116 cells), and lung carcinoma cell line (A549 cells).

#### Impact of sorbate metal complex on human *colon* carcinoma cell line (HCT-116 cells)

**Fig. 6:**
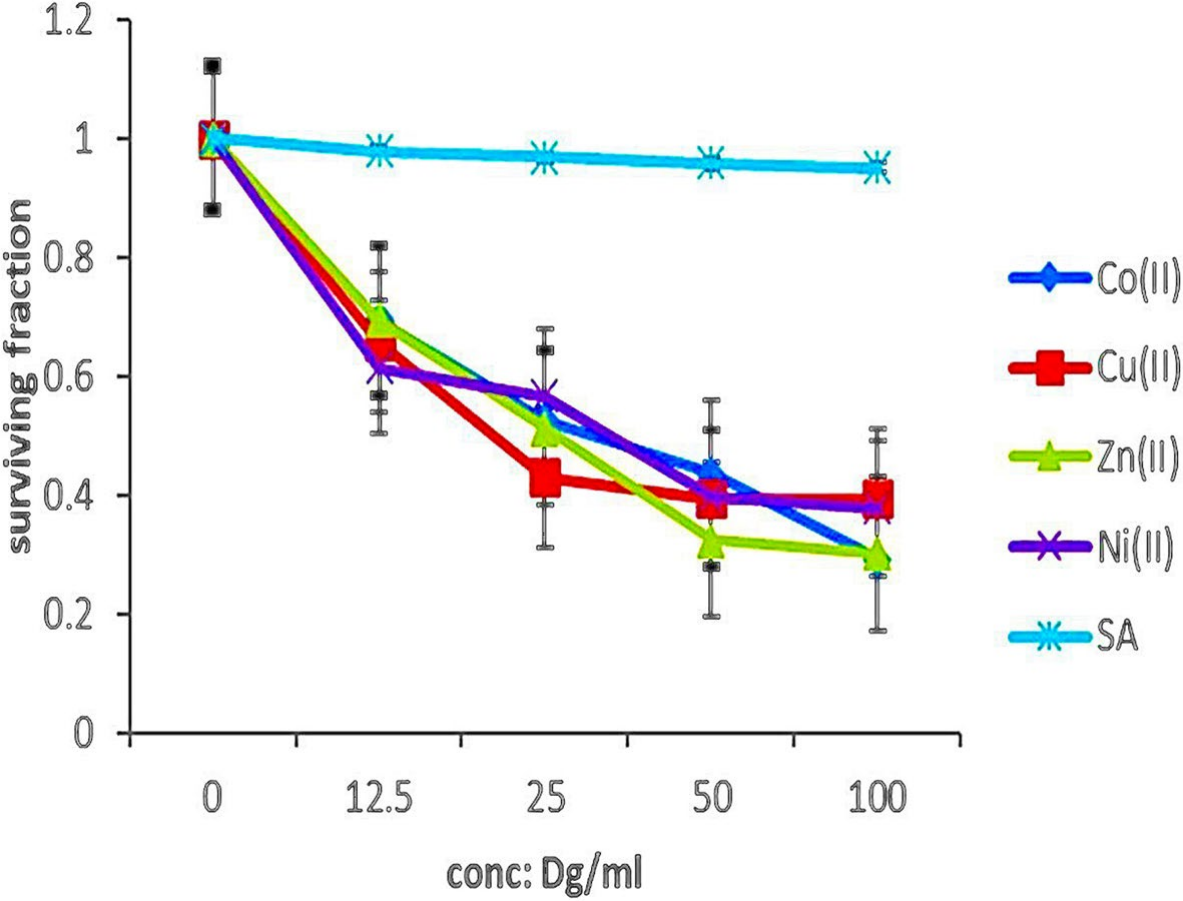
The effect of sorbate metal complexes on the human colon carcinoma cell line (HCT-116)

All metal complexes; Co (II), Cu (II), Zn (II), and Ni (II) affected the human colon carcinoma cell line (HCT-116 cells) with IC_50_ of 3230, 2110, 3730, and 2240 µg/mL for cobalt, copper, zinc, and nickel complexes, respectively. These values indicate the concentration of each complex required to inhibit cell viability by 50% in the HCT-116 cell line. From these results, sorbate metal complexes, particularly the Cu (II) complex, demonstrate promising cytotoxic activity against human colon carcinoma cells, highlighting their potential for further exploration as anticancer agents (Fig. 6**).** Its potency in inducing cytotoxic effects on the human colon carcinoma cell line (HCT-116 cells) signifies its potential as an anticancer agent.

#### Impact of sorbate metal complex on human breast carcinoma cell line (MCF-7 cells) and lung carcinoma cell line (A549 cells)

In contrast to the significant cytotoxic effects observed on the HCT-116 cell line, the tested complexes did not exhibit notable cytotoxicity on the human breast carcinoma cell line (MCF-7 cells) and lung carcinoma cell line (A549 cells). Therefore, these complexes showed negative effects on these cell lines, suggesting a lack of cytotoxicity against breast and lung carcinoma cells.

#### Structure activities relationship regarding to anticancer activities

The analysis of structure–activity relationships (SAR) concerning the anticancer properties of metal-sorbate complexes—zinc, copper, cobalt, and nickel—reveals that the copper sorbate complex displays the lowest IC50 against cancer cells, indicating its exceptional cytotoxicity and efficacy in impeding cancer cell proliferation. This heightened anticancer potency is attributed to the distinctive characteristics of copper ions, which potentially foster stronger interactions with cancer cell constituents, amplify the production of reactive oxygen species (ROS), and disrupt crucial cellular processes. Copper's capacity to undergo oxidation–reduction reactions enables it to induce greater damage to cellular DNA, proteins, and membranes compared to other metals. Conversely, the higher IC50 values for zinc, cobalt, and nickel sorbate complexes imply they are less effective in targeting cancer cells, likely due to differences in their electronic configurations, coordination chemistry, and interactions with cellular targets. This highlights the critical role of metal ion properties in determining the anticancer effectiveness of metal-sorbate complexes, emphasizing copper as particularly potent in this aspect.

## Discussion

The challenge posed by infections caused by biofilm-producing bacteria is a significant threat to public health worldwide [37]. These bacteria exhibit antimicrobial resistance and are adept at evading the immune system, making them difficult to eradicate [38]. Biofilms, a common survival mechanism employed by pathogenic bacteria, render them highly resistant to antimicrobial agents compared to their planktonic counterparts. In addition to posing risks to human health, bacterial biofilms also contaminate food, leading to a loss of nutritional value [39], and can form on the surfaces of medical devices such as catheters and cardiac valves [40]. Given the limitations of conventional antimicrobial drugs and the increasing prevalence of antimicrobial resistance, there is an urgent need for innovative alternative therapies to combat biofilm-associated infections [41, 42]. While plant extracts and essential oils have shown therapeutic potential against resistant pathogens [43, 44], their efficacy in vivo is limited by the need for very high concentrations. Drug repositioning, or the use of existing drugs for new purposes, also has limitations, including a limited number of targets and potential lack of specificity [45]. In this context, we try to pierce the gap between public health crises from both biofilms associated infections in addition to human cell carcinoma and the ineffectiveness of the available drugs. Thus, organometallic complexes such as sorbate metal complexes present a promising alternative for combating biofilm-related infections.

In general, the enhanced biological activity of metal complexes compared to free SA ligands can be attributed to five main factors [46]: (i) the chelation effect, wherein bidentate ligands like SA exhibit greater antimicrobial activity compared to complexes with monodentate ligands; (ii) the nature of the ligands; (iii) the overall charge of the complex; (iv) the nature of the counterion in the case of ionic complexes; and (v) the nuclearity of the metal center in the complex. Chelation also reduces the polarity of metal ions by partially sharing their positive charge with donor groups, enabling potential delocalization of π-electrons within the chelate ring system formed during coordination [47]. This process enhances the lipophilicity of the metal ion, facilitating its permeation through the lipid layer of the membrane [48]. As a result, the molecule's hydrophobic character and liposolubility increase as it crosses the cell membrane of the microorganism, thereby improving the biological utilization rate and activity of the tested SA compounds.

In this study, SA was utilized to form organometallic complexes, which were characterized using various instrumental analyses. Importantly, the antibacterial activities of these complexes were demonstrated, with low minimum inhibitory concentration (MIC) values ranging from 312.5 µg/mL to 625.0 µg/mL. The current study highlights the efficacy of copper sorbate complex as the most influential agent against bacterial isolates, exhibiting large inhibition zones and low MIC values. Interestingly, the combination of organic acids and transition metals showed superior inhibitory effects compared to individual compounds, indicating a synergistic interaction [49]. This observation is consistent with previous reports suggesting that organometallic complexes act as prodrugs, releasing the active metal and enhancing antibacterial activity of this organic compounds [50]. Esterification has been proposed as a strategy to improve the efficacy of SA, with more lipophilic compounds entering cells more easily [51]. Upon dissociation inside microbial cells, sorbate metal complexes release SA, which disrupts microbial cell membranes through various mechanisms, including the presence of sorbate molecules and increased hydrogen ion concentration [52]. Additionally, liberated metals such as copper and silver induce structural changes in bacterial cell walls, interacting with proteins and enzymes and damaging DNA, thereby inhibiting replication [53]. This study also observed that Gram-positive bacteria, such as *B. cereus*, were more sensitive to sorbate metal complexes than Gram-negative pathogens like *E. coli*. This difference in sensitivity can be attributed to the distinct cell wall structures of Gram-positive and Gram-negative bacteria [54]. Gram-positive bacteria lack an outer membrane layer, with peptidoglycan as the main component of the cell wall. In contrast, Gram-negative bacteria possess a complex cell wall structure with a peptidoglycan layer found between the plasma membrane and the lipopolysaccharide outer membrane [55]. The superior activity of sorbate metal complexes against Gram-positive pathogens may be attributed to the hindered penetrability of antibacterial drugs across the outer membrane layer of Gram-negative bacteria. Thus, the easier penetrability of these complexes across Gram-positive cell wall makes them more effective against this bacterial group [56]. Overall, these findings provide valuable insights into the mechanisms underlying the antibacterial activity of sorbate metal complexes and their differential effects on Gram-positive and Gram-negative bacteria.

The study underscores the potential of targeting biofilm formation as a therapeutic strategy against resistant foodborne pathogens, for which conventional antimicrobial drugs often fail to achieve satisfactory treatment outcomes. Combination therapies and modifications of conjugated moieties are promising approaches in this regard [57]. The antibiofilm activities of sorbate metal complexes demonstrated in this study offer new avenues for combating biofilm-related infections. The tested sorbate metal complexes exhibited dose-dependent reductions in microbial adhesion, with the lowest adhesion percentages observed for *B. cereus* and *E. coli* treated isolates at 2.5% and 8.9%, respectively. Particularly, the sorbate copper complex showed superior antibiofilm activities against both bacterial strains. These findings align with previous reports documenting the antibiofilm activities of SA and its salts [58]. Metal complexes have been utilized to prevent biofilm formation by altering microbial cell envelopes [59]. Notably, organo-copper complexes have been shown to exert antibiofilm effects against Gram-negative bacteria by disrupting quorum sensing mechanisms, leading to modified expression of *lasI* and *lasR* genes [60]. Furthermore, organometal compounds have been found to inhibit the adhesion process, which is the initial step in biofilm formation, by preventing cell attachment to surfaces and to each other [61] through the inhibition of polymeric extracellular matrix (ECM) [62]. Moreover, divalent metal cations such as calcium, magnesium, zinc, and manganese can interact with extracellular DNA (eDNA), a key component of the polymeric extracellular matrix (ECM) in biofilms [63]. This interaction may contribute to the inhibition of biofilm production by disrupting the ECM. These previous published reports have corroborated our promising findings concerning the antibiofilm properties of organometallic complexes, including sorbate metal complexes.

The observed synergistic effects between sorbate metal complexes and common antibacterial drugs, as indicated by the fractional inhibitory concentration index (FICI) of less than 0.5, are noteworthy. In cases where bacteria such as *E. coli* and *B. cereus* exhibit resistance to antibiotics like gentamicin, treatment with sorbate metal complexes can sensitize them to the antibiotic's effects. Combination therapies involving metal complexes have been increasingly explored as a solution to combat antimicrobial resistance, particularly in cases where antibiotics alone are ineffective. Metal complexes have been shown to disrupt biofilms through redox processes, making them effective candidates for combination therapies [64, 65]. Previous Study had demonstrated that the antimicrobial activities of sulfa drugs can be enhanced when used in conjunction with organometal compounds [66]. In vivo study on a rabbit keratitis model has shown promising results with combination therapy involving metal complexes and gentamicin, leading to reduced bacterial infiltration and scar size [67]. Furthermore, organometal complexes have been found to restore the antimicrobial activities of traditional antibiotics against resistant strains such as vancomycin-resistant Enterococci. The synergistic effect can be accepted on the base of the interaction of organometal complexes with thiol-containing biomolecules such as microbial enzymes. This interaction on its own had no severe antimicrobial effect; nevertheless, it might increase the antimicrobial activities of other antibiotics [68, 69]. Overall, combination therapies involving sorbate metal complexes hold promise as a strategy to address antimicrobial resistance and combat biofilm-related infections effectively.

The findings regarding the anticancer activities of sorbate metal complexes, particularly the copper (Cu) complex, are significant in the context of colorectal cancer, which accounts for a substantial portion of gastrointestinal cancers worldwide. The rising mortality rates associated with colorectal cancer underscore the urgent need for new and safe treatment methods [70, 71]. The human colon cell line was highly sensitive to four complexes during the present study. The Cu complex was highly effective with an IC50 of 2110 µg/mL, consistent with a previous in vivo study on the human colon adenocarcinoma cell line, which revealed that isopropyl sorbate was significantly more cytotoxic (68% compared to the 28% of SA and potassium sorbate) [72]. A study explained the differences in the cytotoxicity profiles of sorbates by their binding to proteins [73]. The Cu(II)-based complexes were more toxic to cancer cells than normal cells, where the Co(II) chelated with imidazole derivatives was considered significant for finding novel antitumor drugs [74, 75]. The Cu(BrHAP)2 Schiff base compound demonstrated a potent antiproliferative effect in colon cancer cells (HCT-129) with an IC50 value of 2870 µg/mL after 72 h of treatment [76]. Additionally, the copper complex in this study and a previous study exhibited the best antiproliferative activities against MCF-7 human breast cancer cells [77].

The most accepted mechanism of the biological activities of these metal-based compounds is attributed to their higher lipophilicity, which enhances permeability across the cell membrane and other barriers, making them useful for treating tumors and other resistant pathogens. Regarding the safety of these compounds on normal cells, they dissociate inside the target cells, delivering both the toxic metal and the organic compounds [76, 78]. Interestingly, SA and its salts are non-toxic even in large amounts (25 mg/kg per day) and are widely used in the food industry due to their antioxidant, antifungal, and antibacterial properties. Based on toxicological assays, SA and its salts are generally recognized as safe (GRAS). Carbon dioxide and water are the byproducts resulting from the metabolism of SA and its salts [77].

On the other hand, the maximum permissible levels (MPL) of copper, zinc, nickel, and cobalt are 12,000, 20,000, 100, and 210 µg/mL per day, respectively [78]. In this study, the MICs were 625 µg/mL for Co(II), 1250 µg/mL for Cu(II), 1250 µg/mL for Zn(II), and 2500 µg/mL for Ni(II) complexes against *E. coli* isolates. For *B. cereus* isolates, the MICs for Co(II), Cu(II), and Zn(II) were 625.0, 312.5, and 1250.0 µg/mL, respectively. The IC50 for the human colon carcinoma cell line (HCT-116 cells) ranged from 2110 to 3730 µg/mL. In accordance with previously published reports, these doses were safe for clinical use [78]. Overall, these findings indicate that sorbate metal complexes, especially the Cu complex, exhibit promise as potential anticancer agents for treating colorectal cancer (CRC), with a favorable safety level supporting their clinical use.

## Conclusion

There is an urgent need for new therapeutic options to combat antimicrobial resistance and overcome challenges associated with cancer treatment. This study provides persuasive evidence supporting the expansion of research on the potential medical applications of transition metal complexes. The findings underscore the ability of sorbate metal complexes to address a wide range of challenges, including antimicrobial resistance and certain types of cancer such as colon adenocarcinoma, by demonstrating their efficacy alone or in combination with other drugs. This study contributes to expanding therapeutic options for managing life-threatening diseases caused by resistant pathogens or cancer. Moving forward, further research and clinical trials will be essential to fully realize the potential of sorbate metal complexes in clinical medicine and refine their applications for optimal patient outcomes.

Our recommendation is to explore the use of these complexes in combination with existing antibacterial or anticancer drugs to maximize efficacy in managing these diseases. However, it is important to acknowledge the limitations of the study, including the lack of toxicity testing against normal cells and the absence of in vivo studies. Future research should address these limitations to provide a more comprehensive understanding of the clinical applications of sorbate metal complexes. Additionally, investigating the efficacy of these complexes in combination with existing anticancer drugs would further elucidate their therapeutic potential. Overall, this study establishes a foundation for further investigation into sorbate metal complexes as innovative therapeutic agents in combating antimicrobial resistance and cancer.

### Supplementary Information

Supplementary Material 1.

Supplementary Material 2.

Supplementary Material 3.

Supplementary Material 4.

## Data Availability

The datasets used and/or analyses during the current study available from the corresponding author in this published article. The sequence of analyzed 16s rRNA genes were deposited at the NCBI web server (https://www.ncbi.nlm.nih.gov/genbank) under accession numbers OL961694 and MW406995.

## Abbreviations

B: Beef
CRA: Congo red media
DMEM: Dulbecco’s Modified Eagle’s Medium
MBC: Minimum bactericidal concentration
MIC: Minimum inhibitory concentration
MTT: 2-(4,5-Dimethyl-2 thiazolyl) -3,5-diphenyl-2H-tetrazolium bromide
PBS: Phosphate buffer saline
SA: Sorbic acid
