# Supplementary figures and images for: Sorbate metal complexes as newer antibacterial, antibiofilm, and anticancer compounds
Source: BMC Microbiol. 2024 Jul 18;24:262. doi: 10.1186/s12866-024-03370-w (PMC11256447; doi:10.1186/s12866-024-03370-w)

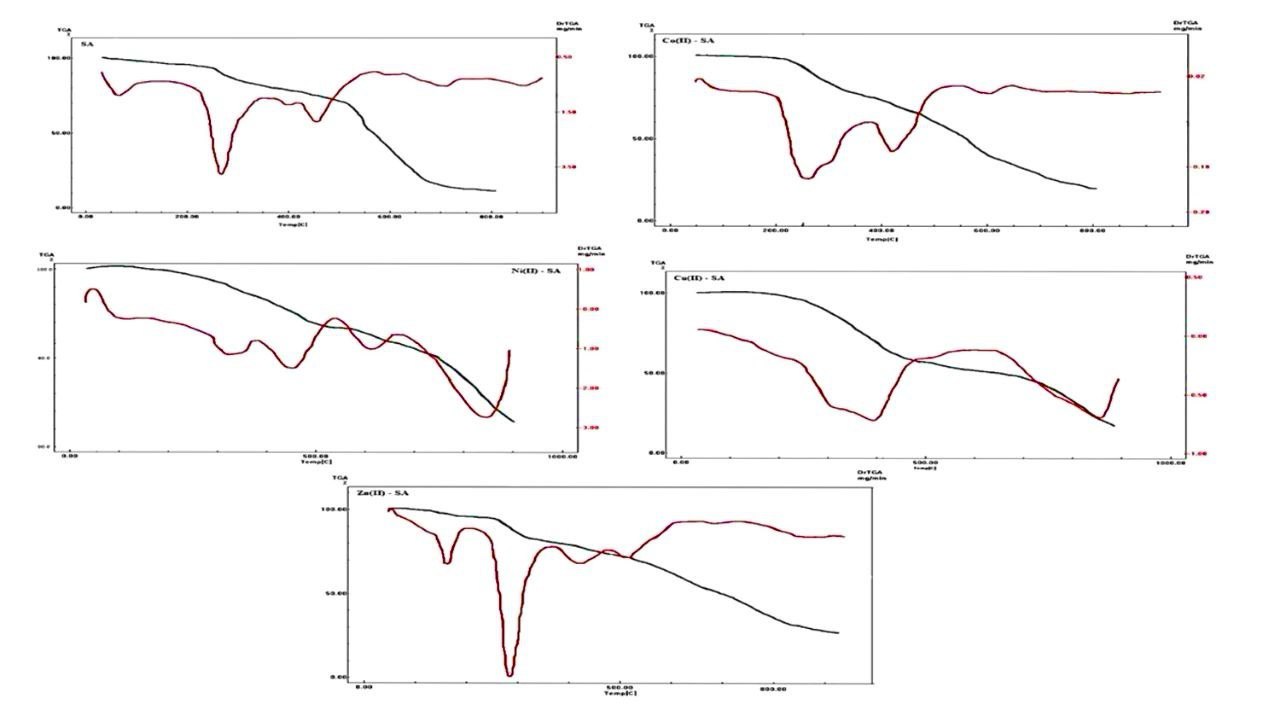

Supplement: Supplementary file 1 — Supplementary Material 1. [file 12866_2024_3370_MOESM1_ESM.jpg]

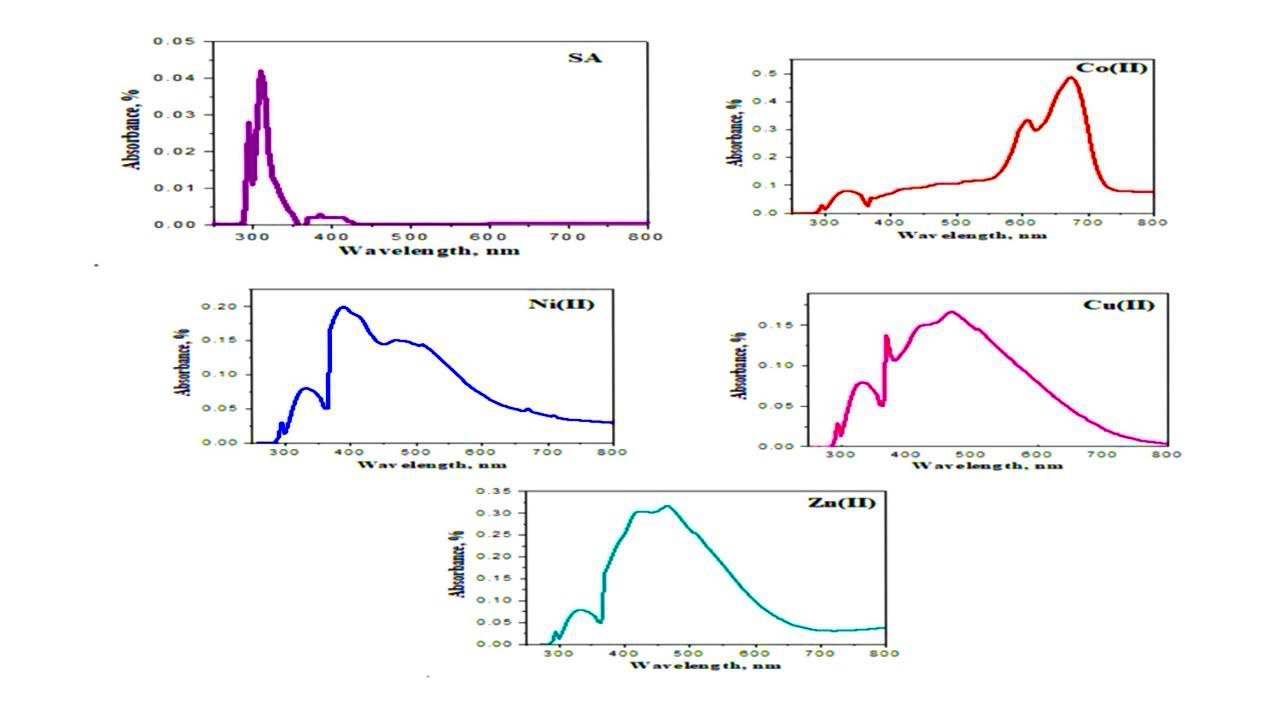

Supplement: Supplementary file 2 — Supplementary Material 2. [file 12866_2024_3370_MOESM2_ESM.jpg]

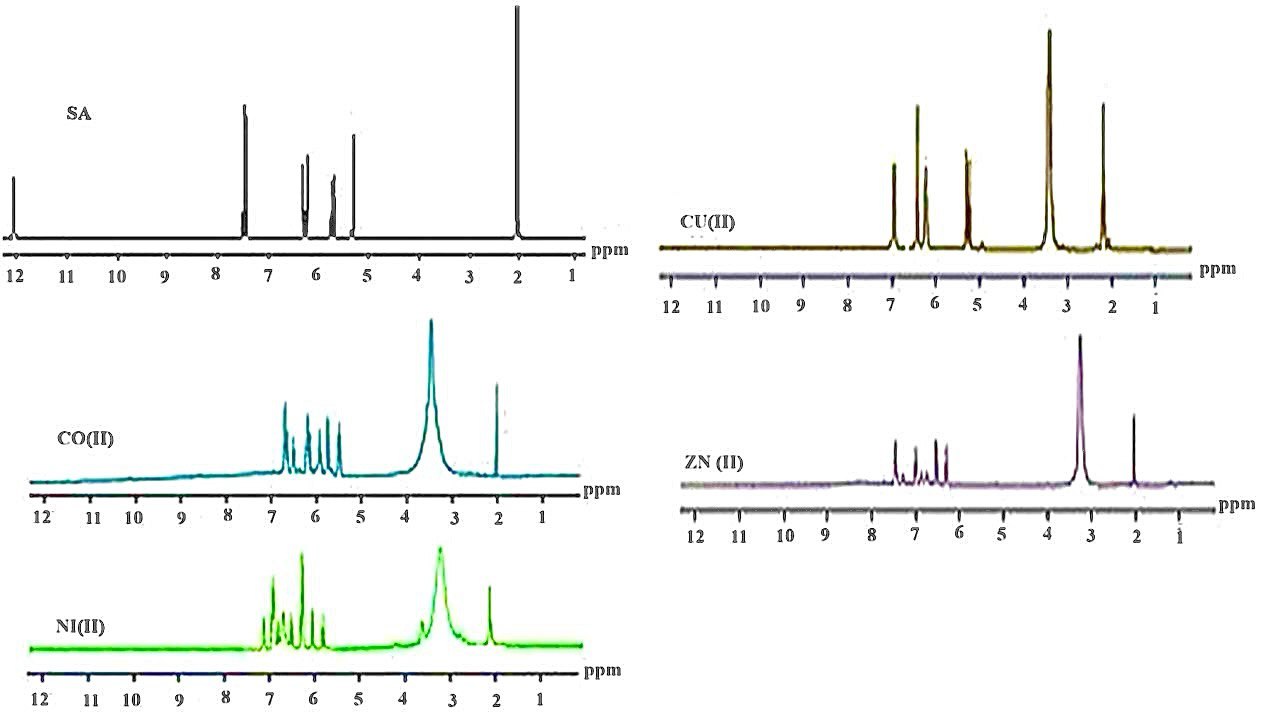

Supplement: Supplementary file 3 — Supplementary Material 3. [file 12866_2024_3370_MOESM3_ESM.jpg]
