# Supplementary material for: Sorbate metal complexes as newer antibacterial, antibiofilm, and anticancer compounds
Source: BMC Microbiol. 2024 Jul 18;24:262. doi: 10.1186/s12866-024-03370-w (PMC11256447; doi:10.1186/s12866-024-03370-w)
Supplement: Supplementary file 4 — Supplementary Material 4. [file 12866_2024_3370_MOESM4_ESM.docx]

**Supplementary tables**

**Table S1:** Elemental analysis and physico-analytical data of sorbic acid (SA) and their complexes:

| **Compounds**  **M.Wt. (M.F.)** | **Yield%** | **Mp/^ο^C** | **Color** | **Found (Calcd.) (%)** | | | **Λ**  **S cm^2^ mol^-1^** |
| --- | --- | --- | --- | --- | --- | --- | --- |
|  |  |  |  | **C** | **H** | **M** |  |
| **SA**  **111.13 (C_6_H_8_O_2_)** | - | 134.53 | White | 63.50  (64.27) | 7.16  (7.19) | - | 12.00 |
| **[Co(SA)_2_(H_2_O)_2_]**  **377.17 (CoC_12_H_18_O_6_)** | 80.03 | 183.22 | White | 38.62  (38.88) | 3.52  (3.74) | 15.43  (15.63) | 19 |
| **[Ni(SA)_2_(H_2_O)_2_]**  **376.93 (NiC_12_H_18_O_6_)** | 77.23 | 288.31 | Pale green | 38.24  (38.12) | 3.74  (3.72) | 15.57  (15.54) | 17 |
| **[Cu(SA)_2_(H_2_O)_2_]**  **321.81 (CuC_12_H_18_O_6_)** | 82.11 | 186.66 | Blue | 44.19  (44.79) | 5.34  (5.64) | 19.16  (19.75) | 10.2 |
| **[Zn(SA)_2_(H_2_O)_2_]**  **323.68 (ZnC_12_H_18_O_6_)** | 85.91 | 210.61 | Off-white | 44.23  (44.53) | 5.08  (5.61) | 20.11  (20.21) | 15.5 |

**Table S2:** Thermo-gravimetric data of Sorbic acid (L) and it^’^s metal complexes:

| **Compounds** | **Decomposition** | **DTG _max_ (^◦^C)** | **% Estimated (calculated)** | **Assignment** |
| --- | --- | --- | --- | --- |
|  |  |  | **Mass loss** | **Lost species** |
| **SA** | First step  Residue | 86,275,444, 740 | 89.99  10.01(10.12) | 2C_2_H_4_+CO_2_  C |
| **[Co(SA)_2_(H_2_O)_2_]** | First step  Second  third step  Residue | 258  425  596  750 | 9.42 (9.54)  70.98 (70.59)  19.60(19.87) | 2H_2_O coordinated  Degradation of ligand moieties  CoO |
| **[Ni(SA)_2_(H_2_O)_2_]** | First step  Second step  Third step  Fourth step  Residue | 210  334,  451,  614 | 9.52 (9.55)  70.71 (70.64)  19.77(19.81) | 2H_2_O coordinated  Degradation of ligand moieties    NiO |
| **[Cu(SA)_2_(H_2_O)_2_]** | First step  Second step  Third step  Fourth step  Residue | 197  394  589  851 | 11.11 (11.18)  64.34 (64.11)  24.55(24.71) | 2H_2_O coordinated  Degradation of ligand moieties  CuO |
| **[Zn(SA)_2_(H_2_O)_2_]** | First step  Second step  Third step  Fourth step  Residue | 151  290  424  526 | 11.00 (11.12)  63.67 (63.74)  25.33(25.14) | 2H_2_O coordinated  Degradation of ligand moieties  ZnO |

**Table S3: Electronic absorption spectral data of SA and their metal complexes**

| **Compounds** | **Peak** | | **Assignment** | **μ_eff_**  **(B.M.)** |
| --- | --- | --- | --- | --- |
|  | **Nm** | **cm^-1^** |  |  |
| **SA** | 293,330,370 | 34130, 30303,27027 | π-π* and n-π* | - |
| **Co(II)** | 295,330,370  472  515  610  675 | 33898,30303,27027  21186  19417  16393  14814 | π-π* and n-π*  ^CT^  ^4^T_1g_(F) → ^4^T_1g_(P)  ^4^T_1g_(F) → ^4^A_2g_(F)  ^4^T_1g_(F) → ^4^T_2g_(F) | 4.81 |
| **Ni(II)** | 295,330,390  470  510  670  710 | 33898,30303,25641  21276  19607  14925  14084 | π-π* and n-π*  ^CT^  ^3^A_2g_(F) → ^3^T_1g_(P)  ^3^A_2g_(F) → ^3^T_1g_(F)  ^3^A_2g_(F) → ^3^T_2g_ | 3.0 |
| **Cu(II)** | 295,330  423,465  510 | 33898,30303  23641,21505  19607 | π-π* and n-π*  ^CT^  ^2^E_g_ → ^2^T_2g_ | 1.83 |
| **Zn(II)** | 295,330  433,475  - | 33898,30303  23094, 21052  - | π-π* and n-π*  ^CT^ | - |

**Table S4: Fractional inhibitory concentration indices (FICI) for the combination of Sorbate Copper (CU) complex with antibiotics such as gentamicin (CN) and imipenem (IMP)**

| **strains** | **Sorbate Copper complex with gentamicin (CN)** | | | | | | | **Sorbate Copper complex with imipenem (IMP)** | | | | | |
| --- | --- | --- | --- | --- | --- | --- | --- | --- | --- | --- | --- | --- | --- |
|  | **MIC**  **CU** | **MIC**  **CU/CN** | **FIC(A)** | **MIC**  **CN** | **MIC**  **CN/CU** | **FIC(B)** | **FICI** | **MIC**  **CU/IMP** | **FIC(A)** | **MIC**  **IMP** | **MIC**  **IMP/CU** | **FIC(B)** | **FICI** |
| B1 | 312.5 | 78.125 | 0.25 | 128 | 8 | 0.0625 | 0.3125 | 39.0625 | 0.125 | 4 | 1 | 0.25 | 0.375 |
| B2 | 625 | 78.125 | 0.125 | 64 | 16 | 0.25 | 0.375 | 156.25 | 0.25 | 2 | 0.25 | 0.125 | 0.375 |
| B3 | 312.5 | 78.125 | 0.25 | 256 | 16 | 0.0625 | 0.3125 | 78.125 | 0.25 | 64 | 8 | 0.125 | 0.375 |
| B4 | 312.5 | 78.125 | 0.25 | 64 | 8 | 0.125 | 0.375 | 78.125 | 0.25 | 4 | 1 | 0.25 | 0.5 |
| E1 | 1250 | 312.5 | 0.25 | 8 | 1 | 0.125 | 0.375 | 156.25 | 0.125 | 1 | 0.25 | 0.25 | 0.375 |
| E2 | 1250 | 312.5 | 0.25 | 1 | 0.25 | 0.25 | 0.5 | 312.5 | 0.25 | 32 | 4 | 0.125 | 0.375 |
| E3 | 312.5 | 78.125 | 0.25 | 2 | 0.5 | 0.25 | 0.5 | 78.125 | 0.25 | 8 | 1 | 0.125 | 0.375 |
| E4 | 1250 | 156.25 | 0.125 | 4 | 0.5 | 0.125 | 0.25 | 156.25 | 0.125 | 2 | 0.5 | 0.25 | 0.375 |
| E5 | 1250 | 312.5 | 0.25 | 2 | 0.5 | 0.25 | 0.5 | 312.5 | 0.25 | 2 | 0.25 | 0.125 | 0.375 |

**Table S5: Fractional inhibitory concentration indices (FICI) for the combination of Sorbate Cobalt (CO) complex with antibiotics such as gentamicin (CN) and imipenem (IMP)**

| **strains** | **Sorbate Cobalt complex with gentamicin (CN)** | | | | | | | **Sorbate Cobalt complex with imipenem (IMP)** | | | | | |
| --- | --- | --- | --- | --- | --- | --- | --- | --- | --- | --- | --- | --- | --- |
|  | **MIC**  **CO** | **MIC**  **CO/CN** | **FIC(A)** | **MIC**  **CN** | **MIC**  **CN/CO** | **FIC(B)** | **FICI** | **MIC**  **CO/IMP** | **FIC(A)** | **MIC**  **IMP** | **MIC**  **IMP/CO** | **FIC(B)** | **FICI** |
| B1 | 625 | 78.125 | 0.125 | 128 | 16 | 0.125 | 0.25 | 156.25 | 0.25 | 4 | 1 | 0.25 | 0.5 |
| B2 | 625 | 156.25 | 0.25 | 64 | 8 | 0.125 | 0.375 | 156.25 | 0.25 | 2 | 0.5 | 0.25 | 0.5 |
| B3 | 1250 | 312.5 | 0.25 | 256 | 32 | 0.125 | 0.375 | 156.25 | 0.125 | 64 | 8 | 0.125 | 0.25 |
| B4 | 625 | 156.25 | 0.25 | 64 | 8 | 0.125 | 0.375 | 78.125 | 0.125 | 4 | 1 | 0.25 | 0.375 |
| E1 | 625 | 78.125 | 0.125 | 8 | 2 | 0.25 | 0.375 | 78.125 | 0.125 | 1 | 0.25 | 0.25 | 0.375 |
| E2 | 625 | 156.25 | 0.25 | 1 | 0.25 | 0.25 | 0.5 | 156.25 | 0.25 | 32 | 4 | 0.125 | 0.375 |
| E3 | 312.5 | 78.125 | 0.25 | 2 | 0.25 | 0.125 | 0.375 | 78.125 | 0.25 | 8 | 2 | 0.25 | 0.5 |
| E4 | 625 | 156.25 | 0.25 | 4 | 0.5 | 0.125 | 0.375 | 78.125 | 0.125 | 2 | 0.5 | 0.25 | 0.375 |
| E5 | 625 | 78.125 | 0.125 | 2 | 0.25 | 0.125 | 0.25 | 78.125 | 0.125 | 2 | 0.5 | 0.25 | 0.375 |

**Table S6: Fractional inhibitory concentration indices (FICI) for the combination of Sorbate Zinc (ZN) complex with antibiotics such as gentamicin (CN) and imipenem (IMP)**

| **strains** | **Sorbate Zinc complex with gentamicin (CN)** | | | | | | | **Sorbate Zinc complex with imipenem (IMP)** | | | | | |
| --- | --- | --- | --- | --- | --- | --- | --- | --- | --- | --- | --- | --- | --- |
|  | **MIC**  **ZN** | **MIC**  **ZN/CN** | **FIC(A)** | **MIC**  **CN** | **MIC**  **CN/ZN** | **FIC(B)** | **FICI** | **MIC**  **ZN/IMP** | **FIC(A)** | **MIC**  **IMP** | **MIC**  **IMP/ZN** | **FIC(B)** | **FICI** |
| B1 | 1250 | 312.5 | 0.25 | 128 | 16 | 0.125 | 0.375 | 156.25 | 0.125 | 4 | 0.5 | 0.125 | 0.25 |
| B2 | 1250 | 156.25 | 0.125 | 64 | 16 | 0.25 | 0.375 | 312.5 | 0.25 | 2 | 0.25 | 0.125 | 0.375 |
| B3 | 1250 | 312.5 | 0.25 | 256 | 32 | 0.125 | 0.375 | 156.25 | 0.125 | 64 | 8 | 0.125 | 0.25 |
| B4 | 1250 | 312.5 | 0.25 | 64 | 4 | 0.0625 | 0.3125 | 312.5 | 0.25 | 4 | 1 | 0.25 | 0.5 |
| E1 | 625 | 156.25 | 0.25 | 8 | 1 | 0.125 | 0.375 | 156.25 | 0.25 | 1 | 0.25 | 0.25 | 0.5 |
| E2 | 1250 | 312.5 | 0.25 | 1 | 0.25 | 0.25 | 0.5 | 312.5 | 0.25 | 32 | 4 | 0.125 | 0.375 |
| E3 | 625 | 78.125 | 0.125 | 2 | 0.5 | 0.25 | 0.375 | 156.25 | 0.25 | 8 | 2 | 0.25 | 0.5 |
| E4 | 1250 | 312.5 | 0.25 | 4 | 0.5 | 0.125 | 0.375 | 312.5 | 0.25 | 2 | 0.5 | 0.25 | 0.5 |
| E5 | 1250 | 312.5 | 0.25 | 2 | 0.5 | 0.25 | 0.5 | 78.125 | 0.0625 | 2 | 0.25 | 0.125 | 0.1875 |

| **strains** | **Sorbate Nickel complex with gentamicin (CN)** | | | | | | | **Sorbate Nickel complex with imipenem (IMP)** | | | | | |
| --- | --- | --- | --- | --- | --- | --- | --- | --- | --- | --- | --- | --- | --- |
|  | **MIC**  **NI** | **MIC**  **NI/CN** | **FIC (A)** | **MIC**  **CN** | **MIC**  **CN/NI** | **FIC**  **(B)** | **FICI** | **MIC**  **NI/IMP** | **FIC (A)** | **MIC**  **IMP** | **MIC**  **IMP/NI** | **FIC (B)** | **FICI** |
| B1 | 5000 | 1250 | 0.25 | 128 | 16 | 0.125 | 0.375 | 625 | 0.125 | 4 | 1 | 0.25 | 0.375 |
| B2 | 5000 | 1250 | 0.25 | 64 | 8 | 0.125 | 0.375 | 1250 | 0.25 | 2 | 0.25 | 0.125 | 0.375 |
| B3 | 5000 | 1250 | 0.25 | 256 | 32 | 0.125 | 0.375 | 1250 | 0.25 | 64 | 16 | 0.25 | 0.5 |
| B4 | 5000 | 625 | 0.125 | 64 | 8 | 0.125 | 0.25 | 625 | 0.125 | 4 | 1 | 0.25 | 0.375 |
| E1 | 2500 | 625 | 0.25 | 8 | 2 | 0.25 | 0.5 | 625 | 0.25 | 1 | 0.25 | 0.25 | 0.5 |
| E2 | 1250 | 312.5 | 0.25 | 1 | 0.25 | 0.25 | 0.5 | 312.5 | 0.25 | 32 | 8 | 0.25 | 0.5 |
| E3 | 2500 | 312.5 | 0.125 | 2 | 0.25 | 0.125 | 0.25 | 312.5 | 0.125 | 8 | 1 | 0.125 | 0.25 |
| E4 | 2500 | 625 | 0.25 | 4 | 0.5 | 0.125 | 0.375 | 625 | 0.25 | 2 | 0.5 | 0.25 | 0.5 |
| E5 | 1250 | 312.5 | 0.25 | 2 | 0.5 | 0.25 | 0.5 | 312.5 | 0.25 | 2 | 0.25 | 0.125 | 0.375 |

**Table S7: Fractional inhibitory concentration indices (FICI) for the combination of Sorbate Nickel (NI) complex with antibiotics such as gentamicin (CN) and imipenem (IMP)**

**Figure S1: Thermal analysis (TGA) of sorbic acid and its complexes.**

**Figure S2: Electronic absorption spectra for SA and their metal complexes.**

**Figure** **S3:** **^1^HNMR spectra for SA and their metal complexes**
